# Central and Peripheral Mechanism of Acupuncture Analgesia on Visceral Pain: A Systematic Review

**DOI:** 10.1155/2019/1304152

**Published:** 2019-05-02

**Authors:** In-Seon Lee, Soyeon Cheon, Ji-Yeun Park

**Affiliations:** ^1^National Center for Complementary and Integrative Health, National Institutes of Health, Bethesda 20892, USA; ^2^Department of Public Health, College of Medicine, National Cheng Kung University, Tainan 70101, Taiwan; ^3^College of Korean Medicine, Daejeon University, Daejeon 34520, Republic of Korea

## Abstract

**Background/Aims:**

Despite the wide use of acupuncture for the management of visceral pain and the growing interest in the pathophysiology of visceral pain, there is no conclusive elucidation of the mechanisms behind the effects of acupuncture on visceral pain. This systematic review aims to provide an integrative understanding of the treatment mechanism of acupuncture for visceral pain.

**Methods:**

Electronic and hand searches were conducted to identify studies that involved visceral pain and acupuncture.

**Results:**

We retrieved 192 articles, out of which 46 studies were included in our review. The results of our review demonstrated that visceral pain behaviors were significantly alleviated in response to acupuncture treatment in groups treated with this intervention compared to in sham acupuncture or no-treatment groups. Changes in the concentrations of *β*-endorphin, epinephrine, cortisol, and prostaglandin E2 in plasma, the levels of c-Fos, substance P, corticotropin-releasing hormone, P2X3, acetylcholinesterase (AchE), N-methyl-D-aspartate (NMDA) receptors, and serotonin in the gut/spinal cord, and the neuronal activity of the thalamus were associated with acupuncture treatment in visceral pain.

**Conclusions:**

Acupuncture reduced visceral pain behavior and induced significant changes in neuronal activity as well as in the levels of pain/inflammation-related cytokines and neurotransmitters in the brain-gut axis. Further researches on the thalamus and on a standard animal model are warranted to improve our knowledge on the mechanism of acupuncture that facilitates visceral pain modulation.

## 1. Introduction

Visceral pain, i.e., pain originating from the thoracic, abdominal, or pelvic regions, is a noticeable symptom associated with various clinical conditions [1, 2]. Visceral pain has different characteristics compared to somatic pain. The former is diffusely localized, not evoked by entire viscera, and rarely linked to actual injuries. Although there has been growing interest in the mechanisms and factors that contribute to the pathogenesis of visceral pain, many researches are still more focused on somatic pain [3]. To date, several underlying causes of visceral pain have been proposed, e.g., visceral hypersensitivity due to sensitized visceral nociceptors/afferent fibers, impairments of the brain-gut axis, referred hyperalgesia from viscerosomatic convergence in the spinal cord and central nervous system (CNS), infections, psychological and genetic factors, and hormonal changes [1, 3, 4]. Moreover, a major advance in the understanding of the central mechanisms and the gut environment (e.g., microbiota) of humans has suggested that the brain-gut axis plays a crucial role in visceral nociception in terms of neuronal/chemical signaling between the brain and the gastrointestinal tract [5, 6].

Acupuncture has been used to treat various pain disorders, including visceral pain, and has shown considerable effects on pain relief with only rare cases of adverse events. Many studies have explored the treatment mechanism of acupuncture for pain relief in general, and it is reported that acupuncture alleviates pain mainly by regulating the levels of endogenous opioids, serotonin, and norepinephrine and by inhibiting visceral nociceptors, inflammatory cytokines, and CNS activation [7–9]. Furthermore, acupuncture can decrease visceral sensitivity [10], activate the enteric nervous system (ENS) [11], and modulate the brain-gut axis [12]. However, the treatment mechanism behind the effects of acupuncture on visceral pain is still unclear [4, 9], partially due to the lack of a systematic approach that encompasses a wide range of evidence from basic and clinical researches to analyze this aspect.

In this review, we aimed to provide an integrative understanding of the mechanisms behind the effects of acupuncture therapy on visceral pain in both human and animal subjects, and we suggest directions that can be adopted for future research.

## 2. Methods

### 2.1. Search Strategy

We searched through electronic records in PubMed, EMBASE, MEDLINE, and the Cochrane Library using the keywords “visceral,” “pain,” “hyperalgesia,” “algesia,” “acupuncture,” “electroacupuncture,” and “acupoint.” The search terms and strategies were modified for each individual database (Table 1). Hand searching was performed by screening the reference lists of articles that met our inclusion criteria. The literature search was completed in September 2017, and our search strategy was in compliance with the Preferred Reporting Items for Systematic Review and Meta-Analyses (PRISMA) guidelines for systematic reviews.

### 2.2. Study Selection

Search results were screened based on the titles and abstracts before full text assessments. We included original studies that investigated the therapeutic effects and/or the mechanisms of acupuncture on visceral pain. Both animal and human studies written in English or Chinese were included. In this review, we considered manual acupuncture (MA), electroacupuncture (EA), transcutaneous electrical nerve stimulation on acupoints (acu-TENS), pharmacopuncture (injection of herbal medicine into acupoints, e.g., sweet bee venom), and laser acupuncture (LA) techniques as the different types of acupuncture.

### 2.3. Risk of Bias Assessment in the Human Studies

Concerning the studies on human subjects, we evaluated the risk of bias associated with each of them using either the revised Cochrane risk of bias tool for randomized trials (RoB 2.0 [13]) or the Risk Of Bias In Nonrandomized Studies (ROBINS-I [14]) based on each study design, with a focus on the pain-related outcomes. More details on the assessments are described in the footnotes of supplementary Tables 1 and 2.

## 3. Results

### 3.1. Search Results

**Figure 1:**
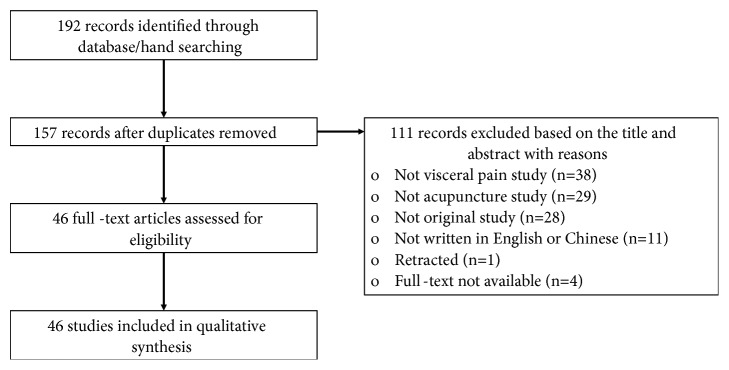
Flow diagram of article inclusion.

Our search strategy resulted in the retrieval of 192 articles in total. Following this, in addition to removing the duplicates (n=35), 111 studies were excluded based on their titles and abstracts. Among the excluded studies, 67 studies were unrelated to visceral pain or acupuncture, 28 studies were not original, and 11 studies were written in languages other than English or Chinese. Further, full texts corresponding to four studies, each published before 1990, could not be obtained, and one study was retracted. Ultimately, 46 articles were included in this review (Figure 1).

### 3.2. Visceral Pain Studies on Humans

#### 3.2.1. Visceral Pain Patients and Healthy Participants

There were seven studies with human subjects. Two studies included irritable bowel syndrome (IBS) patients [15, 16], and one study involved primary dysmenorrhea [17] and another study involved chronic pancreatitis patients [18]. Further, a study by Kotani et al. [19] investigated postoperative visceral pain in patients who underwent upper or lower abdominal surgeries [18], and two studies involved healthy participants [20, 21] (Table 2).

#### 3.2.2. Details of Acupuncture Interventions

**Figure 2:**
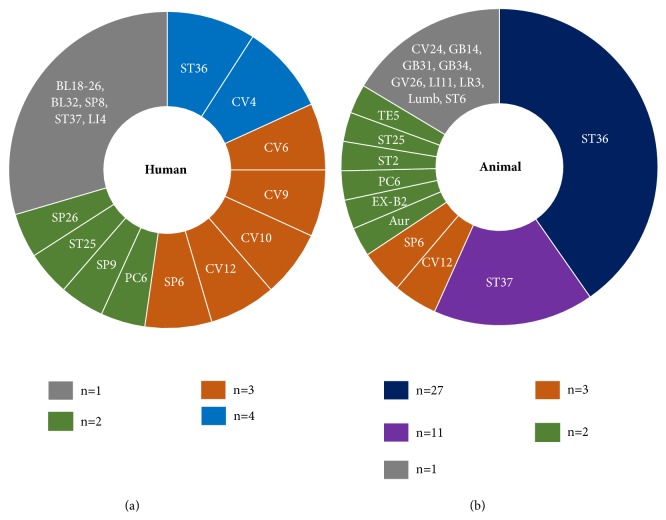
Frequency distribution of acupuncture points in the human and animal studies on visceral pain. Acupuncture points on Stomach Meridian (ST) and Conception Vessel Meridian (CV) are most frequently selected for visceral pain. Aur: auricular acupoints, Lumb: lumbar acupuncture points, n: number of studies reporting the acupuncture points, BL: Bladder Meridian, GB: Gallbladder Meridian, LI: Large Intestine Meridian, LR: Liver Meridian, PC: Pericardium Meridian, SP: Spleen Meridian, and TE: Triple Energizer Meridian.

Among the seven studies conducted on humans, the various types of acupuncture techniques that were used are as follows: MA in four studies [17–19, 21], acu-TENS in three studies [16, 17, 20], and EA in two studies [15, 17]. Most of these studies described the stimulation method that was used, the intensity (frequency, voltage, and amperage), and the locations of acupoints. The acupoints that were used in the human studies are summarized in Figure 2(a).

#### 3.2.3. Outcomes and Results

*(1) Pain Behavioral Outcomes*. In human studies, improvements in the pain behavioral outcomes have been consistently observed after acupuncture treatment. Acupuncture treatment showed significantly greater analgesic effects based on subjective pain ratings than sham acupuncture [16, 18, 19], and the maximum tolerable rectal sensation and distention pressure in IBS patients were significantly increased by acu-TENS compared to sham TENS [20] (Tables 2 and 5).

**Figure 3:**
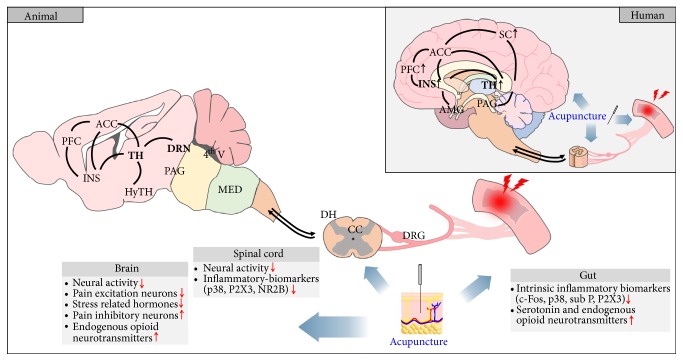
Schematic illustration of functional and metabolic changes (peptides, proteins, and mRNA) of the brain-gut axis by acupuncture in visceral pain studies. In human, acupuncture consistently enhanced the functional activity in the thalamus and insula, the core brain regions of pain processing. In animal, a great variety of metabolic changes have been reported as well as functional neural activities, demonstrating that acupuncture induces a wide range of changes through the brain-gut axis in visceral pain. ACC: anterior cingulate cortex; AMG: amygdala; CC: central canal; DH: dorsal horn; DRG: dorsal root ganglion; DRN: dorsal raphe nucleus; HyTH: hypothalamus; INS: insula; MED: medulla; NR2B: N-methyl-D-aspartate receptor subunit; PAG: periaqueductal gray; PFC: prefrontal cortex; P2X3: P2X purinoceptor 3; SC: somatosensory cortex; sub P: substance P; TH: thalamus; 4th V: fourth ventricle.

*(2) Metabolic Outcomes*. In human studies, the secretion of *β*-endorphin increased after acu-TENS [20], and the levels of adrenal hormones [19] and serum prostaglandin E2 (PGE2) [22] were decreased after MA. The plasma levels of *β*-endorphin were increased after EA [23] (Tables 2 and 5, Figure 3).

*(3) Brain and Brain Stem*. Functional magnetic resonance imaging (fMRI) and electroencephalography (EEG) were used to measure neuronal activity in IBS patients [15] and healthy participants [18, 21]. The fMRI results showed significant increases in the brain activity in the thalamus and insula in the EA group compared to in the sham EA group [15], and there were no significant differences observed in the EEG data between the groups [18, 21] (Tables 2 and 5, Figure 3).

#### 3.2.4. Risk of Bias in the Human Studies

There were three randomized parallel-group trials [15, 19, 20] and two randomized cross-over trials [18, 21] included in our review. One study reported results from two different groups (acupuncture and acu-TENS), and within each group, the assignment of the different interventions to the patients was randomized [17]. Therefore, we treated this study as two randomized cross-over studies and assessed the risk of bias associated with each group separately. Lastly, one study was a nonrandomized trial. The results of the assessments of each domain's risk of bias and the overall risk of bias are presented in Supplementary Tables 1 and 2 for the randomized and nonrandomized studies, respectively.

Out of the seven studies, three studies were classified as having low overall risks of bias. Further, two randomized studies [17, 20] were considered as having high risks of bias due to concerns regarding possible selective reporting. Moreover, there were some concerns regarding three randomized studies [17, 18, 21] with regard to randomization domains because they lacked information regarding either their random sequence generation or their allocation concealment; therefore, we labeled these studies as “some concerns.” Lastly, there were some concerns regarding missing data associated with two studies because they did not provide clear indications on whether the results that were summarized in the texts or figures were from all the study participants.

### 3.3. Visceral Pain Studies on Animals

#### 3.3.1. Visceral Pain Models

We found 39 studies that examined various visceral pain models in animals. Thirty-one studies used rats [10, 23–51], and the remaining used cats [52–54], mice [22, 55, 56], rabbit [57], or dog [58]. To induce visceral pain, mechanical stimulation by colorectal/rectal/gastric distention was most commonly used (n=19) [10, 24, 25, 28, 29, 32, 33, 35–41, 44, 45, 47, 51, 58]. Other methods included splanchnic nerve stimulation [48, 52–54, 57], acetic acid injections [22, 23, 31, 55, 56] (n=5, respectively), somatic and visceral noxious stimuli [49], formalin injection [23], and antimony potassium tartrate injection [50] (Tables 3 and 4).

#### 3.3.2. Details of Acupuncture Interventions

Further, concerning the animal studies, EA was the intervention that was used in most of them [10, 23, 25–29, 31–43, 45, 47–54, 57, 58] (n=32), and the others involved MA [22, 24, 44, 55], LA [30, 46], or pharmacopuncture techniques using bee venom [56] or snake venom [23]. The acupoints that were used in the animal studies are summarized in Figure 2(b).

#### 3.3.3. Outcomes and Results

*(1) Behavioral Outcomes*. In the animal studies, the abdominal withdrawal reflex [25–27, 32–34, 38, 39, 41, 42], abdominal muscle activities, writhing responses in the abdomen and leg, and other pain-related behaviors significantly decreased after administering EA [29, 45, 48, 50] (Tables 3–5).

*(2) Metabolic Outcomes*. Liu et al. found that EA treatment applied at the ST25 and ST37 points reduced the concentrations of 5-hydroxytryptamine (5-HT) when compared to the concentrations observed in the no-treatment visceral hypersensitivity group [27]. Xu et al. also showed increased *β*-endorphin levels in the EA treatment group compared to in the no-treatment formalin injection model [59] (Figure 3).

*(3) Gut*. The concentration levels of serotonin-related factors, hormones, neurotransmitters, and other molecular signaling factors in the colon were observed in the animals before and after acupuncture. The expression levels of serotonin and c-Fos dropped significantly after EA treatment [26, 29]. The expression levels of serotonin transporter (additional water avoidance stress model) [26], c-Fos [23], p38 [23], substance P [23, 46], corticotropin-releasing hormone (CRH) [38], P2X3 receptor [39], acetylcholinesterase (AchE) [46], and the serotonin concentration [27] were significantly lowered after EA. The expression levels of the serotonin transporter (colorectal irritation model) [26], 5-HT4 receptor [27], and leu-enkephalin [46] significantly increased after EA and LA (Tables 3–5, Figure 3).

*(4) Spinal Cord*. In the spinal cords of the studied animals, neuronal activities, glucose metabolic rates, hormones, serotonin-related factors, and pain-related molecules were observed before and after acupuncture. The glucose metabolic rates were significantly decreased in the thoracic dorsal horns and increased in the lumbar dorsal horns and in the periaqueductal gray matter after EA [48]. In the lumbosacral spinal cord, the expression levels of serotonin and c-Fos decreased significantly after EA compared to after sham EA [29]. The c-Fos expression levels also decreased in various regions of the spinal cord after EA [23, 31–33] and MA [55]. The neuronal activity of the dorsal root ganglion significantly decreased after EA [10] and that of the spinal dorsal horn significantly decreased after MA [24]. The expression levels of p38 in the spinal dorsal horn [40], CRH [38], P2X3 receptor [39], and the NR2B subunit of the N-methyl-D-aspartate (NMDA) receptor [42] decreased further after EA (Tables 3–5, Figure 3).

*(5) Brain and Brain Stem*. In the animal studies, the glucose metabolic rates, the levels of serotonin-related neurotransmitters, hormones, and neuronal activity along with the levels of molecular signaling associated with this activity were measured. After EA, the glucose metabolic rates significantly decreased in many regions, e.g., in the thalamus, anterior cingulate cortex (ACC), nucleus accumbens, and somatosensory cortex, and they increased in the nucleus raphe magnus [48]. A significantly increased thalamic neuronal response to colorectal distention was found after EA (versus nonacupoint EA) [28]. CRH concentrations in the hypothalamus were significantly decreased after EA [38], and *β*-endorphin levels in the hypothalamus were significantly elevated after MA [22]. *β*-endorphin and substance P levels in the hypothalamus showed significant increases after EA [45]. The P2X3 receptor expression levels in the prefrontal cortex (PFC) and in the ACC significantly decreased after EA [39] (Tables 3–5, Figure 3).

## 4. Discussion

To our knowledge, this is the first study to systematically review the mechanisms behind the effects of acupuncture on visceral pain studied in both humans and animals through the brain-gut axis. In the 46 included studies in our review, significant improvements in pain-related behaviors were consistently reported in both humans and animals included in the acupuncture treatment groups compared to those included in the sham acupuncture or no-treatment groups. Increased secretion of *β*-endorphin and decreased epinephrine, cortisol, and PGE2 levels may be involved in the acupuncture mechanisms at the systemic level that are responsible for the modulation of visceral pain. Acupuncture treatment reduced c-Fos, substance P, CRH, P2X3, AchE, serotonin, and NMDA receptor expression levels and elevated serotonin receptor/transporter and leu-enkephalin expression levels in the gut and spinal cord. Studies reporting on the functional neuronal activity showed that EA increased the activity of the thalamus more than sham EA during colorectal distention. The effects of acupuncture were blocked by spinalization [24], acute freezing of the spinal cord [44], naloxone [10, 41], capsaicin [31], and infraorbital nerve transection [23, 31, 41], indicating the requirements for acupuncture to have an effect. Moreover, it is conceivable that the mechanisms underlying the effects of EA may differ depending on the frequency of EA administrations. Qi et al. [41] reported that the administration of naloxone inhibited the effects of 2 Hz EA but not those of 100 Hz or 2/100 Hz EA (alternate stimulation at 2 Hz and 100 Hz frequencies).

### 4.1. Plasma

Pain commonly causes hyperactivity of the hypothalamic-pituitary-adrenal system resulting in elevated plasma hormone levels such as those of cortisol, epinephrine, and adrenocorticotropin [60]. In their review, Kotani et al. reported that the increased levels of epinephrine and cortisol in plasma after abdominal surgeries were significantly reduced by MA than by sham acupuncture [19]. Previous studies have also reported that acupuncture attenuated the epinephrine [61] and cortisol [62, 63] levels in plasma. With regard to how EA normalized the increased low-frequency/high-frequency ratio caused due to stress in the functional dyspepsia model [43], the results we have discussed demonstrate the modulatory effects of acupuncture on acute stress caused by visceral pain via sympathetic activity and the hypothalamic-pituitary-adrenal axis.

### 4.2. Gut

Since visceral pain originates in the gastrointestinal tract and its peripheral regions, changes in the immune and nervous systems and changes in the microbial environment of the gut have been investigated. *β*-endorphin is an endogenous opioid neuropeptide that has an analgesic effect [64], and PGE2 is a hormone-like substance that participates in a wide range of bodily functions such as muscle activity, blood pressure control, pain sensation, and inflammation [65]. Neurotransmitter substance P, the levels of which decreased after acupuncture [23, 46], is also involved in inflammation and plays an important role in the mechanisms of acupuncture related to pain modulation [66]. Serotonin, another important neurotransmitter in the CNS and gastrointestinal tract, regulates various functions of the digestive tract. Serotonin and its receptors are extensively distributed in the myenteric nerve plexus and participate in the regulation of abnormal symptoms in the GI tract [67, 68]. It has been reported that the levels of serotonin and the activities of various types of its receptors are found to be increased in the intestinal mucosa of visceral pain patients [69]. Serotonin induces and maintains visceral hyperactivity by modulating the activation of the transient receptor potential vanilloid 1 (TRPV1) [70]; thus, serotonin receptor agonists or antagonists have been investigated for the treatment of IBS patients [71, 72]. In our review, three studies reported that serotonin levels were lower in the EA groups compared to in the sham EA [29] or no treatment groups [27] or in the model groups [26] while 5-HT4 receptor and serotonin transporter levels significantly increased after EA treatment in visceral pain models [26, 27] and decreased after EA treatment in visceral pain groups that received additional stress conditions [26]. These results suggest that changes in neuropeptide concentrations may vary depending on the type of visceral pain model and the stress levels and that acupuncture has bidirectional effects on diverse systems in order to modulate pain.

### 4.3. Spinal Cord

The spinal cord is the first region in which incoming pain signals are transmitted to the central nerves. The spinal cord receives sensory information from the whole body and transmits this information to several regions of the brain that are responsible for processing pain [73]. In this review, the studies reported that the action potential, c-Fos, serotonin, p38, and NMDA receptor levels in the spinal dorsal horn were all significantly decreased after EA [10, 29]. C-Fos is commonly used as a marker to measure neuronal activity [74]; thus, increased c-Fos activation in response to the spinal cord signals represents the excitement of the CNS, similar to the effects of an increased action potential. Serotonin, p38, and NMDA receptors are involved in the development of visceral pain [75] and the central sensitization of visceral pain [76]. In addition, the effects of acupuncture were inhibited by spinalization [24]. These results indicate that the spinal cord plays a critical role in the analgesic mechanism of acupuncture for visceral pain. However, conflicting results among electrical or chemical measurements associated with various nuclei hinder the development of an integrative understanding.

### 4.4. Brain

A set of brain regions, collectively called the “visceral pain network,” are the core of the perception and modulation of internal and external stimuli in the gut [77, 78]. An fMRI study found that the activities of the thalamus and insula significantly increased during EA treatment compared to during sham EA stimulation when the participants underwent visceral distention [15]. Complementary to fMRI measurements in humans, which could not distinguish between the excitatory and inhibitory neurons, Sun et al. [49] reported that EA significantly reduced the discharge of pain excitation neurons and enhanced the discharge of pain inhibition neurons in the thalamus of rats. This bidirectional influence of acupuncture (to inhibit or enhance neuronal activity) might have led to the inconsistent results in the evaluations of the thalamic activity during/after acupuncture in the animal studies. In other visceral pain network regions, P2X3 receptor activity was decreased more in the PFC and ACC in the EA group (versus no-treatment IBS model group) during colorectal distention [39]. The thalamus receives signals from its periphery and relays them to the hypothalamus, insula, PFC, and motor and somatosensory cortex—the so-called visceral pain network [79–81]. The insula integrates sensory information received from visceral and motor activities with inputs from the limbic system [77, 80]. The thalamus is mainly associated with the first-order processing of sensory information, whereas the PFC, insula, and ACC tend to be associated with the higher-order processing of cognitive evaluation, attention, sensory-motor integration, and affective responses [77]. This observation implies that acupuncture treatment influences higher-level cortical activity along with the primary visceral sensory processing regions.

### 4.5. Quality Assessment of the Included Studies

We only assessed the quality of the human studies and found that only three studies were classified as having low overall risks of bias while two studies were considered as having high risks of bias. A few assessment tools have been developed for assessing the quality of animal studies, but they are not widely accepted nor validated in the field yet. Moreover, since most of the animal studies included in our review did not report on having employed ways to minimize the risk of bias, such as the blinding of the outcome assessor or randomization methods, we could only assume that they were at high risks of bias.

It is also important to assess the reporting quality of the acupuncture interventions. There are well-known guidelines, the STRICTA guidelines (STandards for Reporting Interventions in Clinical Trials of Acupuncture [82, 83]), for clinical studies that use acupuncture treatment. However, there are no such guidelines that have been designed specifically for reporting acupuncture interventions in animal studies. For these reasons, we evaluated the quality of reporting on acupuncture interventions according to the STRICTA guidelines in a separate paper [84]. To improve our understanding of the underlying mechanisms of acupuncture that are involved in the treatment of visceral pain, studies conducted according to well-validated guidelines with detailed reports on the acupuncture intervention employed are warranted.

### 4.6. Summary

Based on these results, we found that acupuncture induces analgesic effects on visceral pain via multiple pathways from the peripheral organs (gut) to the CNS (brain). Visceral organs are where visceral pain occurs, and acupuncture directly regulates visceral pain by reducing the levels of intrinsic inflammatory biomarkers and increasing the levels of serotonin and endogenous opioid neurotransmitters. The neural signals induced by acupuncture are also transmitted to the brain through the spinal cord, and it attenuates the peripheral neural activity and concentrations of the inflammatory-biomarkers such as p38, P2X3, and NR2B in the spinal cord. In the brain, acupuncture attenuates the levels of neural activity and pain excitation neurons in the thalamus and reduces stress-related hormone levels in the hypothalamus, which suggests that the neural and hormonal changes in the thalamus and hypothalamus are involved in the pain modulatory effects of acupuncture on visceral pain. Moreover, acupuncture induces an increase in the levels of *β*-endorphin and pain inhibitory neurons that are also related to pain inhibition.

With this review, we were able to present the broad outline of the acupuncture signal-transduction system from the gut to the CNS, but the acupuncture signaling pathways from the spinal cord to the intestine or the gut-brain signal-transduction system are still unclear. Further experimental studies are needed to elucidate the entire signaling mechanism of acupuncture from the peripheral to the central organs.

## 5. Conclusion

This review summarizes the findings from previous studies associated with the neural and chemical changes that take place through the brain-gut axis in both humans and animals in order to reveal the underlying mechanisms behind the effects of acupuncture treatment on visceral pain. The results of this review demonstrated significant improvements in visceral pain following acupuncture treatments. However, achieving an integrative understanding of the mechanism of acupuncture on visceral pain remains a long-term endeavor. High heterogeneity among the included studies (various visceral pain conditions and models along with diverse outcome measures and heterogeneous results) and the lack of detailed descriptions outlining the treatment methods also raise concerns.

In future studies, the thalamus and the brain-gut axis could be considered as targets or markers of the visceral pain that is modulated by acupuncture. Furthermore, studies on changes in the levels of neurotransmitters or neuropeptides in the gut and the brain may improve our knowledge of visceral pain modulation by acupuncture treatment.

## Figures and Tables

**Table 1:** Search terms used in each database.

Database	Search terms
PUBMED	(acupuncture [MeSH Terms] OR acupuncture [All Fields] OR acupoint [All Fields] OR electroacupuncture [MeSH Terms] OR electroacupuncture [All Fields]) AND (“visceral pain” [MeSH Terms] OR “visceral pain” [All Fields] OR (visceral [All Fields] AND (pain [All Fields] OR pain [MeSH Term])) OR (visceral [All Fields] AND (hyperalgesia [All Fields] OR hyperalgesia [MeSH Terms])))
EMBASE	visceral AND (“pain”/exp OR pain OR “hyperalgesia”/exp OR hyperalgesia OR algesia) AND (“acupuncture”/exp OR acupuncture OR “electroacupuncture”/exp OR electroacupuncture OR acupoint)
MEDLINE	(visceral and (pain or hyperalgesia or algesia) and (acupuncture or electroacupuncture or acupoint)).mp.
Cochrane Library	visceral AND (pain OR hyperalgesia OR algesia) AND (acupuncture OR electroacupuncture OR acupoint)

**Table 2:** Overview of visceral pain studies on humans.

Author (year)	Participants group:n(f)/ mean age	Acupuncture groups		Control groups		Outcomes	Results
		Acupuncture	acupoints number, duration	Control	acupoints number, duration		
(1) Thomas et al. (1995) [17]	Primary dysmenorrhea Acu: 17(17)/30.4 Con: 12(12)/27.8	(a) MA (b) EA (2Hz) (c) EA (100Hz) (d) Periosteal stimulation (e) acu-TENS (2Hz) (f) acu-TENS (100Hz)	(a), (b), (c), (d): BL32, CV4, SP6, 9 (e), (f): Spinous processes Thoracic 10-Lumbar 1 2, 20min	(g) Sham TENS (ns)	Spinous processes Thoracic 10-Lumbar 1 2, 20min	(1) Blood loss (2) Vomiting (3) Work hours lost (4) Tablet intake (5) Subjective assessment (6) Total pain	Within group (4), (5), (6) (a), (c), (d) improved (5), (6) (b) improved (4), (5), (6) (e) improved
(2) Kotani et al. (2001) [19]	(1) Upper abdominal surgery (A) 50(21)/52 (C) 48(18)/55 (2) Lower abdominal surgery (B) 39(12)/55 (D) 38(13)/55	(A) Patient 1 +MA (B) Patient 2 +MA	(A) BL18, 19, 20, 21, 22, 23, 24 (B) BL20, 21, 22, 23, 24, 25, 26 1, 4 days	(C) Patient 1+sham MA (ni) (D) Patient 2+sham MA (ni)	(C) BL18, 19, 20, 21, 22, 23, 24 (D) BL20, 21, 22, 23, 24, 25, 26 1, 4 days	(1) Incisional, visceral pain (2) Drowsiness (3) Pruritus (4) Nausea/Vomiting (5) Adequacy of pain treatment (6) Morphine intake (7) Adrenal hormone	Within group (1) (A), (C) improved (6) (A), (B), (C), (D) decreased Between groups (1), (4), (6), (7) (epinephrine, cortisol) (A)<(C), (B)<(D)
(3) Xing et al. (2004) [16]	IBS +rectal distention 7(6)/44	(a) acu-TENS (5Hz, 250ms)	(a) ST36, PC6 1, -	(b) TENS (5Hz, 250ms)	(b) non-acupoint 1, -	(1) Rectal tone (2) Rectal compliance (3) Rectal perception of gas, pain, desire to defecate	Within group (1) (A), (B) decreased (3) (A) decreased Between groups (3) (A)<(B)
(4) Chu et al. (2012) [15]	IBS +rectal distention (A) 15(7)/42.3 (B) 15(8)/44.2	(A) EA (10Hz, 0.5ms, 60v)	(A) ST36, 37, SP6 2, 30min	(B) Sham EA (ns)	(B) ST36, 37, SP6 2, 30min	(1) fMRI-rectal distention (2) fMRI-A or B +rectal distention (3) fMRI-rectal distention after (2) (4) fMRI-A or B (5) Rectal sensation	Within group (1) (A), (B): ACC, pgCC, PFC, TH, INS, cerebellum, Temp (A): (1)<(2) pgCC, ACC, PFC, SC, INS, Temp (1)<(3) PFC, SC, Temp (3)<(2) ACC, PFC, TH, INS, Temp (B): (2)>(1) ACC, PFC, SC (3)>(1) PFC, Temp, cerebellum (2)>(3) Temp (3)>(2) PFC Between groups (A)>(B): (2)>(1) in TH, INS Correlation between (5) and brain activation in hypothalamus, TH, INS
(5) Leung et al. (2013) [20]	Healthy +rectal distention (A) 20(12)/53.4 (B) 20(12)/53.9	(A) acu-TENS (2Hz, 0.2ms)	(A) LI4, PC6, ST36 1, 45min	(B) Sham TENS (ns)	(B) LI4, PC6, ST36 1, 45min	(1) Tolerance to rectal sensation (2) Rectal distention pressure (3) Beta-endorphin	Between groups (1), (2), (3) (B)<(A)
(6) Juel et al. (2016) [21]	Healthy +rectal distention 15(8)/27.6	(a) MA	(a) CV4, 6, 7, 9, 10, 12, ST25, 26, 37, LI4 +non-acupoints 1, 30min	(b) Sham MA (ni)	(b) ST37, LI4 1, 30min	(1) Rectal distention volume (2) Rectal pain (3) EEG	Within groups (1) (A), (B) increased
(7) Juel et al. (2017) [18]	Chronic pancreatitis 15(7)/61.8	(a) MA	(a) CV4, 6, 9, 10, 12, ST25, ST36, SP6, 8, 9, 15+non-acupoints 1, -	(b) Sham MA (ni)	(b) CV4, 6, 9, 10, 12, ST25, ST36, SP6, 8, 9, 15+non-acupoints 1, -	(1) Reduced pain score (2) EEG	Between groups (1) (B)<(A)

Group written in lowercase letters (e.g., (a), (b), and (c)): different treatments in the same population, unless stated otherwise; Group written in capital letters (e.g., (A), (B), and (C)): different treatments in different population

Acu: acupuncture group; ACC: anterior cingulate cortex; BL: Bladder Meridian; Con: control group; CV: Conception Vessel Meridian; EA: electro-acupuncture; EEG: electroencephalography; f: female; fMRI: functional magnetic resonance imaging; IBS: irritable bowel syndrome; INS: insula; LI: Large Intestine Meridian; MA: manual acupuncture; n: number; ni: not inserted; ns: not stimulated; PC: Pericardium Meridian; PFC: prefrontal cortex; pgCC: perigenual cingulate cortex; SC: somatosensory cortex; SP: Spleen Meridian; ST: Stomach Meridian; Temp: temporal lobes; TH: thalamus; (acu-)TENS: Transcutaneous electrical nerve stimulation (on acupoints); min: minutes; ms: milliseconds; v: volts.

**Table 3:** Visceral hypersensitivity models in animal studies (visceral hypersensitivity and CRD models).

Author (year)	Model (animal, gender, number)	Acupuncture groups		Control groups		Outcomes	Results
		Acupuncture	acupoints number, duration	Control	acupoints number, duration		
(1) Cui et al. (2005) [25]	Visceral hypersensitivity+CRD (SD rat, m, various)	(A) Model+EA (4/100Hz, 1mA)	(A) ST36, 37 7, 30min	(B) Control (C) Model (D) Model+Sham EA (ns)	(A) ST36, 37 7, 30min	(1) AWR (2) Activity of rectus abdominis	Between groups (1), (2) (A)<(C), (B)<(C)
(2) Tian et al. (2006) [26]	(1) Colorectal irritation-induced visceral hypersensitivity (SD rat, m, 6/group) (2) Model 1+stress	(A) Model 1+EA (2Hz, 0.3mA) (B) Model 2+EA (2Hz, 0.3mA)	(A), (B) ST36, SP6 1, 30min	(C) Control (D) Model 1 (E) Model 2	-	(1) AWR (2) Pain threshold pressure (3) Fecal pellet (4) 5-HT4a receptor in colon (5) Serotonin transporter in colon	Between groups (1) (A), (C)<(D) (2) (D)<(A), (C) (3) (B), (C), (D)<(E) (4), (5) (B)<(A), (C) (5) (D)<(C)
(3) Liu et al. (2009) [27]	Visceral hypersensitivity (SD rat, m, 8/group)	(A) Model+EA (2/100Hz, 0.2–0.6ms, 1mA)	(A) ST25, ST37 7, 20min	(B) Control (C) Model	-	(1) AWR (2) Concentration of 5-HT (3) Concentration of 5-HT3R (4) Concentration of 5-HT4R	Between groups (1), (2) (A), (B)<(C) (4) (C)<(A), (B)
(4) Xu et al. (2009) [10]	Visceral hypersensitivity +CRD (SD rat, m, 6-25/group total 74)	(A) Model+EA (2/100Hz, 0.1ms, 1mA) (B) Model+EA+SAL (same as above)	(A), (B) ST36 5, 40min	(C) Control (D) Model (E) Model +Sham EA (ns) (F) Model+EA+NAL	(E), (F) ST36 5, 40min	(1) VMR (2) Membrane potential of DRG neuron (3) Rheobase of DRG neuron (4) Action potential of DRG neuron	Within group (1) (D): increased (1) (A), (B): decreased after treatment (effect blocked in (F)) Between groups (2) (A)<(E), (C)<(D) (3) (D)<(C), (E)<(A) (4) (A), (C)<(D)
(5) Wu et al. (2010) [29]	Visceral hypersensitivity +CRD (SD rat, m, 8/group)	(A) Model+EA (10Hz, 0.18ms, ~3mA)	(A) ST36 3, 20min	(B) Control (C) Model+Sham EA (ns)	(B) ST36 3, 20min	(1) Pain threshold to CRD (2) VMR (3) 5-HT in colon, spinal cord, brainstem (4) c-Fos in colon, spinal cord, brainstem	Between groups (1) (C)<(A), (B) (2), (3), (4) (A), (B)<(C) (except (3) in colon) (B)<(A) (in 40, 60, 80mmHg)
(6) Qi et al. (2012) [32]	Visceral hypersensitivity +CRD (SD rat, m, 8/group)	(A) Model+EA (5/100Hz)	(A) ST36, 37 4, 30min	(B) Control (C) Model (D) Model+sham EA (ns)	(D) ST36, 37 4, 30min	(1) Pain threshold pressure (2) AWR (3) c-Fos in RVM (4) N-methyl-D-aspartate receptor 1 in RVM	Within group (2) (A): decreased after treatment Between groups (1) (C)<(A), (B) (2) (B)<(C) (3) (A)<(C) (4) (A), (B)<(C)
(7) Qi et al. (2012) [33]	Visceral hypersensitivity +CRD (SD rat, m, 8/group)	(A) Model+EA (5/100Hz)	(A) ST36, 37 4, 30min	(B) Control (C) Model (D) Model+sham EA (ns)	(D) ST36, 37 4, 30min	(1) AWR (2) c-Fos in spinal cord	Between groups (1), (2) (A), (B)<(C)
(8) Zhou et al. (2012) [34]	Stress-induced visceral hypersensitivity (SD rat, m, 5-8/group total 94)	(A) Model+EA (2/100Hz, 0.1ms, 1mA)	(A) ST36 1, 30min	(B) Model (C) Control+ Sham EA (ns) (D) Control+EA (E) Model+Sham EA (F) Model+EA (G) Model+EA+SAL (H) Model+EA+NAL (I) Model+EA+NAL methiodide	(C)-(E), (H)-(J) ST36 (F) BL43 1, 30min	(1) VMR (2) AWR (3) Distention pressure threshold	Within group (1), (2) (B): increased (vs baseline) (3) (B): decreased (vs baseline) Between groups (1) (A)<(E), (G)<(I) (40, 60, 80mmHg) (2) (A), (C)<(E) (D)<(E) (60, 80mmHg) (G)<(H)
(9) Weng et al. (2015) [39]	Visceral hypersensitivity +CRD (SD rat, m, 8/group)	(A) Model+EA (2/100Hz, 2mA)	(A) ST25, 37 7, 20min	(B) Control (C) Model	-	(1) AWR (2) P2X3 receptor in colonic, DRG, spinal cord, PFC, ACC	Between groups (1), (2) (A), (B)<(C)
(10) Qi et al. (2016) [41]	Visceral hypersensitivity+CRD (SD rat, m, various/group total 184)	(A) Model+EA (2Hz, 1-3mA) (B) Model+EA (100Hz, 1-3mA) (C) Model+EA (2/100Hz, 1-3mA)	(A)-(F) ST36, 37 1, 10min	(D) Model+EA (2Hz, 1-3mA)+NAL (E) Model+EA (100Hz, 1-3mA)+NAL (F) Model+EA (2/100Hz, 1-3mA)+NAL (G) Model+sham EA (ns) (H) Control	(D)-(G) ST36, 37 1, 10min	(1) AWR (2) Activity of rectus abdominis	Within group (1), (2) (A)-(C), (E), (F): increased after CRD, decreased after treatment (D), (G): increased after CRD
(11) Liu et al. (2017) [42]	Visceral hypersensitivity (SD rat, m, 8/group)	(A) Model+EA (5/25Hz, 1mA)	(A) ST36, 37 4, 30min	(B) Control (C) Model (D) Model+sham EA (ns)	(C) ST36, 37 4, 30min	(1) AWR (2) NR2B in spinal cord	Between groups (1) (A)<(C) (2) (A), (B)<(C), (D)
(12) Zhou et al. (2017) [43]	Visceral hypersensitivity +restraint stress (SD rat, m, 16/8)	(a), (b) Model+EA (100Hz, 1mA)	(a), (b) ST36 (a) 1, 90min (b) 1, 30min	(C) Control (d) Model (e) Model+sham EA (ns) (f) Model+propranolol (g) Model+phentolamine (h) Model+SAL	(d) ST36 1, 30min	(1) Activity of acromiotrapezius (2) ECG	Within group (2) (d): LF/HF increased after restraint stress Between groups (1) during gastric distention: (C), (a), (b)<(d); (f), (g)<(h) (2) LF/HF, LF: (a)<(d)

Group written in lowercase letters (e.g., (a), (b), and (c)): different treatments in the same population, unless stated otherwise. Group written in capital letters (e.g., (A), (B), and (C)): different treatments in different population.

ACC: anterior cingulate cortex; AWR: abdominal withdrawal reflex; BL: Bladder Meridian; CRD: colorectal distention; DRG: dorsal root ganglion; EA: electro-acupuncture; ECG: Electrocardiography; f: female; HF: high frequency heart rate variability; LF: low frequency heart rate variability; m:male; mA: milliampere; min: minutes; ms: milliseconds; NR2B: N-methyl-D-aspartate receptor subunit NR2B; NAL: naloxone; ns: not stimulated; PFC: prefrontal cortex; RVM: rostral ventromedia medulla; SAL: saline; SD: Sprague Dawley; ST: Stomach Meridian; VMR: visceral motor response (reflex); 5-HT: 5-hydroxytryptamine (serotonin).

**Table 4:** Visceral hypersensitivity models in animal studies (other models).

Author (year)	Model (animal, gender, number)	Acupuncture groups		Control groups		Outcomes	Results
		Acupuncture	Acupoints number, duration	Control	Acupoints number, duration		
(1) Du et al. (1976) [52]	Splanchnic nerve stimulation (cat, -,-)	(A) Model+EA (25/70/100Hz)	(A) GB31, 34, LI11, TE5 -	-	-	(1) VSR	Within group (1) (A): inhibited
(2) Zhang et al. (1989) [53]	Splanchnic nerve stimulation (cat, -, total 35)	(A) Model+EA (5Hz)	(A) PC6 1,3-5min	(B) Model+Morphine (C) Model+EA+NAL	(C) PC6 1, 3-5min	(1) C-CEPs (2) A-CEPs	Within group (1) (A), (B): reduced amplitudes (2) (A), (B): reduced amplitude of late component
(3) Guoxi (1991) [54]	Splanchnic nerve stimulation (cat, m, total 219)	(A) Model+EA	(A) ST36 -	-	-	(1) Electrical activities in thalamus	Within group (1) (A): inhibited in 45 out of 48 neurons
(4) Shu et al. (1994) [48]	Splanchnic nerve stimulation (Wistar rat, m, 3/4/4)	(A) Model+EA	(A) ST36, SP6 -	(B) Control (C) Model	-	(1) Pain threshold (2) Glucose metabolic rate	Within group (1) (A): increase after treatment Between groups (2) (A)<(C): TDH, LC, ventral PAG, CPC, HL, Hippo, CP, NRD, NCS, TPV, ACC, accumbens, NSL, SC (C)<(A): LDH, NRM, NRG, PAG (B)<(C): NRM, PGL, LC, NRG, HA, CCGM
(5) Cai et al. (1994) [57]	Splanchnic nerve stimulation (rabbit, m/f, 8/4/6/6/7/9/6/6)	(A) Model+EA (25Hz)+SAL	(A) EX-B2 (T12,L1,L2) 1, 30min	(B) Model+SAL (C) Model+ MCP (D) Model+EA (25Hz)+MCP (E) Model+EA+MCP+SAL (F) Model+EA+MCP+APO (G) Model+EA+MCP+SKF38393 (H) Model+EA+MCP+LY171555	(D)-(H) EX-B2 1, 30min	(1) Pain threshold (2) DA in CSF (3) DOPAC in CSF (4) HVA in CSF	Within group (1) (A), (C), (D), (F): elevated compared to baseline (4) (A), (C): elevated compared to baseline Between groups (1) (A), (C)<(D); (B)<(C); (F), (G), (H)<(E) (4) (A),(C)>(B)
(6) Kwon et al. (2001) [56]	Acetic acid injection (ICR mice, m, 10-20/group)	(A) Model+BV (B) Model+BV+NAL (C) Model+BV+Yo	(A) CV12 1, -	(D) Model+BV (E) Model+SAL	(D) non-acupoint 1, -	(1) Abdominal stretches (2) c-Fos in spinal cord, NTS	Between groups (1) (A), (D)<(E) (2) (A)<(E)
(7) Yu et al. (2008) [55]	Acetic acid injection (mice, m/f, 12/group)	(A) WT Model+MA (B) Connexin 43 gene knockout HT Model+MA	(A),(B) CV12, ST36 1, 30min	(C) WT control (D) HT control (E) WT model (F) HT model	- -	(1) c-Fos in the spinal dorsal horn	Between groups (1) (A)<(E), (A)<(B)
(8) Yu et al. (2008) [22]	Acetic acid injection (mice, m/f, 18/group)	(A) Model+WT MA (B) Connexin 43 gene knockout HT ++Model+MA	(A),(B) CV12, ST36 1, 30min	(C) WT control (D) HT control (E) WT model (F) HT model	- -	(1) Latency of writhing response (2) Number of writhing response (3) *β*-endorphin in hypothalamus (4) Plasma PGE_2_	Between groups (1), (3) (A)>(E), (A)>(B) (2), (4) (A)<(E), (A)<(B)
(9) Liu et al. (2010) [23]	Acetic acid injection (SD rat, m/f, 6/group)	(A) Model+EA (2/20Hz) (B) Model+EA+Snake venom (C) Modal+EA+SAL	(A)-(C) ST2 1, 20 min	(D) Control (E) Model (F) Model+EA+ION transection	(F) ST2 1, 20 min	(1) Abdominal muscular contractions (2) c-Fos expression in the NTS (3) c-Fos expression in the PTN	Between groups (1), (2) (A),(E)>(D); (A),(B),(C)<(E), (A)<(F) (3) (A),(B),(C)>(E), (A),(B),(C)>(F)
(10) Liu et al. (2011) [31]	Acetic acid injection (SD rat, -, -)	(A) Model+EA (2/20Hz, 1.0mA) (B) Model+EA (2/20Hz, 2.0mA) (C) Model+ EA (2/20Hz, 4.5mA) (D) EA (2/20Hz) (E)-(G) Model+EA (2/20Hz)	(A)-(E) ST2 (F) GB14 (G) ST6 1, 20min	(H) Control (I) Model (J) Model+Sham EA (ns) (K) Model+EA (2/20Hz)	(J) ST2 (K) non-acupoint 1, 20min	(1) c-Fos (2) Abdominal contractions	Within group (1) (D): in the NTS, CPTN, PTN, postrema, DMN of the vagus, RF (blocked by infraorbital nerves transaction pretreatment in PTN) (1) (B), (C), (E), (G), (K): inhibited in NTS (1) (B)-(E), (G), (K): increased in PTN (1), (2) (E): infraorbital nerves transaction and capsaicin pretreatment inhibited the effect of EA on reduced (1) and (2) Between groups (2) (D), (E), (G), (H), (K)<(I) (B), (C)<(A), (I), (K)
(11) Gong et al. (1992) [50]	Antimonium potassium tartrate injection (Wistar rat, m/f, 15/10/7/-/12/8/9/8/6/6/10/8/15/15)	(A) Model+EA (45/12.5Hz) (B) Model+EA (45/12.5Hz) +electrical stimulation of PVN	GV26, CV24 1, 20min	(C) Control (D) Model (E) Model+Sham EA (ns) (F) Model+EA+sham stimulation of PVN (G) Model+EA+lesion of PVN (H) Model+EA+sham lesion of PVN (I) Model+EA+vasopressin antagonist (J) Model+EA+SAL (K) Model+EA+vasopressin antiserum (L) Model+EA+normal serum (M) Model+EA+vasopressin antagonist (N) Model+EA+SAL	(E)-(N) GV26, CV24 1, 20min	(1) Writhing response	Between groups (1) (A)<(E), (B)<(F), (G)>(H), (I)>(J), (K)>(L)
(12) Xu et al. (2010) [59]	Formalin injection (SD rat, m, 8/group)	(A) EA (20Hz, ~1mA) (B) Model+EA (20Hz, ~1mA)	(A), (B) EX-B2 (L3, 6) 1, 20min	(C) Control (D) Model	-	(1) Visceral pain behavior (2) p38 in colon, spinal dorsal horn (3) c-Fos in colon, spinal dorsal horn (4) Plasma *β*-endorphin (5) SP in colon	Between groups (1) (B)<(D) (2), (3), (5) (B), (C)<(D) (4) (C)<(D)<(B)
(13) Rong et al. (2005) [44]	CRD (SD rat, m, total 67)	(A) Model+non receptive field MA (2-3Hz) (B) Model+receptive field MA	(A) ST36 (contr) (B) ST36 (ips) 1, 30s	(C) Model (D) Model+non receptive field pinch (E) Model+ receptive field pinch (F) Model+hot water on tail (non receptive filed) (G) Model+ non receptive field MA+acute freeze of spinal cord (H) Model+acute freeze of spinal cord	(G) ST36 (contralateral) 1, 30s	(1) Electrical activities of spinal dorsal horn WDR neurons of L1-L3	Within groups (1) (A), (D): decreased (B), (G), (H): increased Between groups (1) (A),(D)<(C); (A)<(G)
(14) Rong et al. (2005) [24]	CRD (SD rat, -, 17/17/9/15/9)	(A), (B) Model+MA (2Hz)	(A) ST36 (contr) (B) ST36 (ips) 1, 30s	(C) Pinch (contr) (D) Pinch (ips) (E) Model	-	(1) Electrical activities in spinal dorsal horn	Between groups (1) (A), (C)<(E) ((A)<(E) effect blocked after spinalization)
(15) Zhang et al. (2009) [28]	CRD (SD rat, m, 58)	(a), (b) Model+EA (2Hz, 1mA)	(a) ST36 (contr) (b) ST36 (ips) 1, 320s	(c) Model (d) Model+EA (2Hz, 1mA, 0.5ms) (e) Model+EA (100Hz, 1mA, 0.5ms)	(c)-(e) center of receptive field 1, 320s	(1) Electrical activities in thalamus	Between groups (1) (d), (e)<(c) (e)<(d) (d)<(a), (b)
(16) Chen et al. (2010) [51]	CRD (Wistar rat, m, 9/group)	(A) Model+EA (B) Model+EA (C) Model+EA (2/15Hz, 2mA)	(A) ST36 (B) PC6 (C) LR3 1, 15min	(D) Control (E) Model+Sham EA (non-acupoint, 2/15Hz, 2mA)	(E) non-acupoint 1, 15min	(1) MAP (2) HR (3) HRV HF (4) LF (5) LF/HF	Within group (2) (A), (B) decreased Between groups (1) (A),(B)<(C),(D),(E) (4) (A),(B),(C)<(D) (5) (A),(B)<(D)
(17) Liu et al. (2014) [35]	CRD (SD rat, m, 62)	(a)-(c) Model+EA (10Hz, 2mA)	(a) ST36 (b) PC6 (c) Auricular acupoint (heart) 1, 30s	-	-	(1) Electrical activities in NTS	Within group (1) (a), (b), (c): reduced in 5-10 neurons and increased in 2 neurons among 21 excited neurons by CRD reversed in 9-15 neurons among 24 inhibited neurons by CRD
(18) Yu et al. (2014) [36]	CRD (SD rat, m, 10-11/group)	(a) Model+EA (15Hz, 1.5mA) (b) Model+EA (15Hz, 6mA) (c) Model+EA (15Hz, 4mA)	(a)-(c) ST36, 37 2, 30s	(d) Model	-	(1) Electrical activities of wide dynamic range neurons (2) Electrical activities of SRD neurons	Within group (1) (a), (b): increased after treatment and CRD (d): increased (2) (c), (d): significant responses (a), (b): increased after CRD
(19) Li et al. (2014) [37]	CRD (SD rat, f, 8/7/7/7)	(A) Model+auricular EA (25Hz, 0.8mA)	(A) Auricular acupoints (stomach, small intestine) 1, 30min	(B) Control (C) Model (D) Model+sham EA	(D) no vagal innervation points 1, 30min	(1) VMR (2) mRNA of 5-HT1a receptor in colon (3) mRNA of 5-HT1a receptor in raphe nuclei	Within group (1) (C): increased Between groups (1) (B)<(A), (C), (D) (1), (2) (A)<(C) (3) (A)<(C), (D)
(20) Yu et al. (2014) [47]	CRD (SD rat, m, 22)	(a) preEA+Model+EA (15Hz, 1mA) (b) preEA+Model+EA (15Hz, 4mA) (c) preEA+Model+EA (15Hz, 7mA) (d) preEA+Model+EA (15Hz, 10mA)	(a)-(d) ST36 (ips) 2, 30s	-	- -	(1) Electrical activities of WDR in lumbar spinal cord	Within group (1) (a), (b), (c), (d): increased
(21) Liu et al. (2015) [38]	CRD (SD rat, m, 8/group)	(A) Model+EA (2/100Hz)	(A) ST37 7, 20min	(B) Control (C) Model (D) Model+sham EA (ns)	(D) ST37 7, 20min	(1) AWR (2) CRH in colon (3) CRH in spinal cord, hypothalamus (4) mRNA of CRH in colon, spinal cord, hypothalamus	Between groups (1) (A), (B)<(C); (A)<(D) (2) (A), (B), (D)<(C) (3) (A), (B)<(C),(D) (except (A)<(D) in the spinal cord) (4) (A), (B)<(C); (B)<(D)<(C) (only in hypothalamus)
(22) Rong et al. (2015) [40]	CRD (SD rat, m, 26)	(a) Model+EA (20Hz)	(A) ST36, 37 2, 30s	(b) Model	-	(1) Discharge frequency of VPL neurons in thalamus	Within group (1) (a): increased after CRD (b): increased
(23) Iwa et al. (2005) [58]	Rectal distension (Dog, f 4, 6/group)	(A) Model+EA (10Hz)	(A) ST36 1, 30min	(B) Model+EA (C) Model+EA+NAL (D) Model+EA+NAL methiodide	(B) BL21 (C), (D) ST36 1, 30min	(1) Arterial blood pressure	Within group (1) (A), (D): decreased after treatment
(24) Lin et al. (2009) [45]	Gastric distension (SD rat, m, 10/group)	(A) Model+EA (2Hz, 1-3mA) (B) Model+EA (100Hz, 1-3mA)	(A),(B) ST36 7, 30min	(C) Control (D) Model (E) Sham model	- -	(1) Pain score (2) *β*-endorphin in hypothalamus (3) SP in hypothalamus	Within group (1) (A), (B) decreased Between groups (1) (B)>(A)>(D)>(C),(E) (2),(3) (A)>(B)>(D)>(C),(E)
(25) Sun et al. (1991) [49]	Somatic and visceral noxious stimuli (Wistar rat, m/f, 23/28/-/16)	(A) Model+EA (6v, 8Hz)	(A) ST36 1, 15min	(B) Model (C) Model+Morphine (D) Model+Morphine+NAL	- -	(1) Discharge of PEN in VPN (2) Discharge of PIN in VPN	Within group (1) (A): reduced (2) (A): enhanced
(26) Lorenzini et al. (2010) [30]	Cystitis (SD rat, m, total 48)	(A) Cystitis+PWL	(A)-(F) ST36, TE5 Various	(B) Cystitis	-	(1) Urinary bladder weight, mucosal erosion, ulceration, edema, petechial hemorrhages, thickness of bladder wall (2) Visceral pain behavior	Within group (1), (2) (A), (B): increased
(27) Yang et al. (2010) [46]	Visceral traction (SD rat, m, 10/group)	(A) Model+ LA (650nm, 10mW)	(A) ST36 1, 30min	(B) Sham model (C) Model (D) Model+Moxa	(D) ST36 1, 30min	(1) Pain score (2) Systolic pressure (3) AChE (4) SP (5) LEK (6) Positive index of c-Fos protein (7) Positive index of GFAP	Between groups (1), (3) (A),(B),(D)<(C) (2) (A),(B)<(C) (4) (B)<(A)<(D)<(C) (5) (A)>(B),(C),(D) (6), (7) (B)<(A),(D)<(C)

Group written in lowercase letters (e.g., (a), (b), and (c)): different treatments in the same population, unless stated otherwise. Group written in capital letters (e.g., (A), (B), and (C)): different treatments in different population.

ACC: anterior cingulate cortex; A-CEPs: cortical evoked potentials of A-fibers; AChE: acetylcholinesterase; APO: apomorphine; AWR: Abdominal withdrawal reflex; BL: Bladder Meridian; BV: bee venom; C-CEPs: cortical evoked potentials of C-fibers; CCGM: centralis corpus geniculatum medialis; contr: contralateral; CP: caudate putamen; CPTN: caudal spinal trigeminal nucleus; CRD: colorectal distension; CRH: corticotropin-releasing hormone; CSF: cerebrospinal fluid; CV: Conception Vessel Meridian; DA: dopamine; DMN: dorsal motor nucleus; DOPAC: dopaceticacid; EA: electro-acupuncture; f: female; GB: Gall Bladder Meridian; GFAP: glial fibrillary acidic protein; GV: Governing Vessel Meridian; HA: hypothalamic arcuatus; HF: high frequency heart rate variability; Hippo: hippocampus; HL: habenulae lateralis; HR: heart rate; HRV: heart rate variability; HT: heterozygote; HVA: homovanillic acid; ION: infraorbital nerve; ips: ipsilateral; IT: intrathecal injection; L: lumbar vertebrae; LA: laser acupuncture; LC: locus coeruleus; LDH: lumbar dorsal horns; LEK: leu-enkephalin; LF: low frequency heart rate variability; LI: Large Intestine Meridian; LR: Liver Meridian; m:male; MA: manual acupuncture; mA: milliampere; MAP: mean arterial pressure; MCP: metoclopramide; min: minutes; Moxa: moxibustion; mRNA: messenger ribonucleic acid; ms: milliseconds; NAL: naloxone; NCS: nucleus centralis superior; NRD: nucleus raphe dorsalis; NRG: nucleus reticular gigantocellularis; NRM: nucleus raphe magnus; ns: not stimulated; NSL: nucleus septal lateralis; NTS: nucleus tractus solitarii; PAG: periaqueductal gray; PC: Pericardium Meridian; CPC: centromedian-parafascicula; PEN: pain-excitation neurons; PGE2: prostaglandin E2; PGL: paragigantocellularis lateralis, PIN: pain-inhibitory neurons; PTN: paratrigeminal nucleus; PVN: paraventricular nucleus; PWL: pulsed wave laser; RF: reticular formation; SAL: saline; s: seconds; SC: somatosensory cortex; SD: Sprague Dawley; SP: substance P; SRD: subnucleus reticularis dorsalis; ST: Stomach Meridian; T: thoracic vertebrae; TDH: thoracic dorsal horns; TE: Triple Energizer Meridian; TPV: thalamic posterior ventralis; VMR: visceral motor response (reflex); VPL: ventralis posterior lateralis; VPN: ventral posterolateral nucleus; VSR: viscerosomatic reflex discharges; WDR: wide dynamic range; WT: wild type; Yo: yohimbine; 5-HT: 5-hydroxytryptamine receptor.

**Table 5:** Summary of significant results from the included studies.

Outcomes		Within acupuncture group (baseline vs post-treatment)	vs. sham acupuncture group	vs. no treatment group
Behavioral	Human	Intake of analgesics ↓ [17, 19]	Analgesic effect ↑ [16, 18, 19]	
	Animal	Pain score ↓ Pain threshold ↑ [45, 48]	Pain threshold ↑ [32]	Pain score/behavior ↓ [46, 59] Pain threshold ↑ [29]
		Abdominal withdrawal reflex ↓ [32, 41]	Abdominal withdrawal reflex ↓ [34, 38]	Abdominal withdrawal reflex ↓ [25–27, 33, 38, 39, 42]
		Abdominal muscle activity ↓ [31, 56]	Abdominal muscle activity ↓ [10, 29, 34, 37]	Abdominal muscle activity ↓ [23, 25, 43]
			Writhing response ↓ [50]	Writhing response ↓ [22]

Metabolic	Human		*β*-endorphin ↑ [20] Adrenal hormones ↓ [19]	
	Animal			*β*-endorphin ↑ [59] Serotonin/5-HT 3 receptor ↓ [27]

Gut	Animal		c-Fos ↓ [29]	Serotonin ↓ 5-HT4 receptor ↑ [27] Serotonin transporter ↑ [26] c-Fos ↓ p38 ↓ [59] AchE ↓ Leu-enkephalin ↑ [46] Substance P ↓ [46, 59] CRH ↓ [38] P2X3 ↓ [39]

Spinal cord	Animal	Neural activity in wide dynamic range neurons ↑ [44, 47]/ ↓ [44]	Serotonin and c-Fos in superficial dorsal horn ↓ [29]	Metabolic rate of glucose in thoracic dorsal horns ↓/lumbar dorsal horns ↑ [48] Neural activity in wide dynamic range neurons ↓ [44] Neural activity in spinal dorsal horn ↓ [24] Action potential in dorsal root ganglion ↓ [10] c-Fos in spinal dorsal horn ↓ [55] P38 in spinal dorsal horn↓ [59] NR2B in spinal dorsal horn, central canal region ↓ [42] CRH ↓ [38] P2X3 ↓ [39]

Brain/brain stem	Human	Perigenual cingulate/prefrontal cortex, temporal lobes, insula, somatosensory cortex ↑ [15]	Thalamus, insula ↑ [15]	
	Animal	Neural activity in subnucleus reticularis dorsalis ↑ [36] Discharge frequency of thalamus ↑ [40] Activity of thalamus ↓ [54] Pain inhibitory neurons ↑, pain excitation neurons ↓ in ventral posterolateral nucleus (thalamus) [49] Homovanillic acid in the fourth ventricle ↑ [57]	Neuronal response to colorectal distention in thalamus ↑ [28] Serotonin and c-Fos in dorsal raphe nucleus ↓ [29]	Glucose metabolic rate in ventral periaqueductal gray, nucleus centralis superior ↓/ periaqueductal gray, gigantocellular reticular nucleus ↑ [48] c-Fos, glial fibrillary acidic protein in medulla ↓ [46] c-Fos in nucleus tractus solitarii ↓ [56] in paratrigeminal nucleus ↑ [23] NMDA receptor 1 in rostral ventromedial medulla ↓ [32] CRH in hypothalamus ↓ [38] P2X3 receptor in prefrontal and anterior cingulated cortex ↓ [39] Homovanillic acid in the fourth ventricle ↑ [57] *β*-endorphin in hypothalamus ↑ [22, 45] Substance P in hypothalamus ↑ [45]

AChE: acetylcholinesterase; CRH: corticotropin-releasing hormone; NMDA: N-methyl-D-aspartate; NR2B: N-methyl-D-aspartate receptor subunit NR2B; 5-HT: 5-hydroxytryptamine receptor; P2X3: P2X purinoceptor 3.

## Supplementary Materials

Supplementary Materials Two supplementary tables reporting the risk of bias of included clinical studies regarding the quality of the included studies. We assessed the risk of bias with RoB 2.0 tool for randomized controlled trials (n=6, supplementary Table 1) and ROBINS-I tool for non-randomized clinical trial (n=1, supplementary Table 2).
