# Supplementary material for: Central and Peripheral Mechanism of Acupuncture Analgesia on Visceral Pain: A Systematic Review
Source: Evid Based Complement Alternat Med. 2019 May 2;2019:1304152. doi: 10.1155/2019/1304152 (PMC6521529; doi:10.1155/2019/1304152)
Supplement: Supplementary Materials — Two supplementary tables reporting the risk of bias of included clinical studies regarding the quality of the included studies. We assessed the risk of bias with RoB 2.0 tool for randomized controlled trials (n=6, supplementary Table 1) and ROBINS-I tool for non-randomized clinical trial (n=1, supplementary Table 2). [file 1304152.f1.docx]

Supplementary Table 1: Results of risk of bias assessment of randomized controlled clinical trials by the RoB 2.0 tool*

|  |  | **Study Design** | **Risk by Domains** | | | | | **Overall**  **Risk of Bias** |
| --- | --- | --- | --- | --- | --- | --- | --- | --- |
|  |  |  | **Bias arising from the randomization process** | **Bias due to deviations from intended interventions** | **Bias due to missing outcome data** | **Bias in the measurement of the outcome** | **Bias in selection of the reported result** |  |
| **Thomas et al.** | **Acupu-ncture** | Randomized cross-over^†^ | Some concerns | Low | Low | Low | High | High |
|  | **TENS** | Randomized cross-over^†^ | Some concerns | Low | Low | Low | High | High |
| **Kotani et al.** | | Randomized controlled parallel | Low | Low | Low | Low | Low | Low |
| **Chu et al.** | | Randomized controlled parallel | Low | Low | Low | Low | Low | Low |
| **Leung et al.** | | Randomized controlled parallel | Low | Low | Low | Low | High | High |
| **Juel et al. 2016** | | Randomized controlled cross-over | Some concerns | Low | Some concerns | Low | Low | Some concerns |
| **Juel et al. 2017** | | Randomized controlled cross-over | Some concerns | Low | Some concerns | Low | Low | Some concerns |

*RoB 2.0 tool consists of five domains including (1) bias of the randomization process; (2) bias due to deviations from the intended interventions; (3) bias due to incomplete outcomes; (4) bias in the measurement of the outcomes; and (5) selection bias of the reported outcomes. Same domains were applied for randomized cross-over trials, but with more tailored questions intended specifically for the cross-over design [86]. Risk of bias judgement for each domains and an overall risk of bias judgment were made based on the full guidance document published by the tool authors [87], and marked as ‘low risk of bias,’ ‘some concerns,’ or ‘high risk of bias.’

^†^The study reported results from two different groups (acupuncture and TENS), and within each group, entry to different interventions was randomized. Therefore, we treated this study as two randomized cross-over studies and assessed the risk of bias separately.

RoB: Risk of Bias; TENS: Transcutaneous electrical nerve stimulation

Supplementary Table 2: Results of risk of bias assessment of non-randomized controlled clinical trial by the ROBINS-I tool*

| **Studies** | **Risk by Domains** | | | | | | | **Overall**  **Risk of Bias** |
| --- | --- | --- | --- | --- | --- | --- | --- | --- |
|  | **Bias due to confounding** | **Bias in selection of participants** | **Bias in classification of interventions** | **Bias due to deviations from intended interventions** | **Bias due to missing data** | **Bias in measurement of outcomes** | **Bias in selection of the reported result** |  |
| **Xing et al.** | Low | Low | Low | Low | Low | Low | Low | Low |

*ROBINS-I tool for the non-randomized studies evaluates risk of bias in seven domains: (a) bias due to confounding; (b) bias in the selection of the participants; (c) bias in the classification of the interventions; (d) bias due to deviations from the intended interventions; (e) bias due to incomplete outcomes; (f) bias in the measurement of the outcomes; and (g) selection bias of the reported outcomes. Reaching a domain level and an overall judgement about risk of bias was also based on the detailed guidance [88], and the available response options were ‘low risk of bias,’ ‘moderate risk of bias,’ ‘serious risk of bias,’ ‘critical risk of bias,’ and ‘no information.’

ROBINS-I: Risk Of Bias In Non-randomized Studies
